# Risk Factors for Hot Flashes Associated with Androgen Deprivation Therapy in Japanese Patients with Prostate Cancer

**DOI:** 10.31662/jmaj.2025-0481

**Published:** 2026-02-27

**Authors:** Momoko Sawada, Ryo Inose, Kosuke Okasho, Yuichi Muraki, Shuji Kono

**Affiliations:** 1 Department of Pharmacy, Federation of National Public Service Personnel Mutual Aid Associations Hirakata Kohsai Hospital, Osaka, Japan; 2 Laboratory of Clinical Pharmacoepidemiology, Kyoto Pharmaceutical University, Kyoto, Japan; 3 Department of Urology, Federation of National Public Service Personnel Mutual Aid Associations Hirakata Kohsai Hospital, Osaka, Japan

**Keywords:** prostate cancer, androgen deprivation therapy, hot flashes, beta-blockers

## Abstract

**Introduction:**

Androgen deprivation therapy plays a central role in the treatment of prostate cancer. However, its side effect of hot flashes reduces the quality of life of patients. The risk factors for hot flashes in Japanese patients have been explored in a few studies, but the effects of medications for comorbid conditions have not been fully investigated. This study aimed to determine the risk factors for hot flashes associated with androgen deprivation therapy in Japanese patients with prostate cancer.

**Methods:**

We conducted a single-center retrospective cohort study in patients with prostate cancer who received luteinizing hormone-releasing hormone agonists or antagonists at our institution from January 2013 to December 2023. The patient demographics, comorbidities, and association between concomitant medications and hot flash onset were investigated using multivariate logistic regression analysis.

**Results:**

Of the 180 eligible patients, 99 (55.0%) experienced hot flashes. Multivariate logistic regression analysis revealed that younger age was associated with an increased risk of hot flashes (odds ratio: 1.056, 95% confidence interval: 1.004-1.110, p = 0.034). Concomitant atrial fibrillation (odds ratio: 0.257, 95% confidence interval: 0.071-0.928, p = 0.038) and beta-blocker use (odds ratio: 0.265, 95% confidence interval: 0.086-0.816, p = 0.021) were associated with a decreased risk of developing hot flashes.

**Conclusions:**

Younger age is a risk factor for hot flashes during androgen deprivation therapy in Japanese patients with prostate cancer. Beta-blockers may be promising for preventing or treating hot flashes.

## Introduction

Prostate cancer was the second most common cancer among men globally and the most common in Japan in 2022 ^(1), (2)^. However, it has a relatively low mortality rate and is associated with long-term survival potential ^(3)^. Japan is a rapidly aging society, with approximately 30% of the population aged 65 years or older ^(4)^. The incidence of prostate cancer increases markedly with age ^(5)^, and the number of patients with prostate cancer is increasing in Japan ^(6)^. Androgen deprivation therapy (ADT) plays a central role in prostate cancer treatment, either in combination with radiation therapy for high-risk localized disease or as an essential therapy for advanced disease ^(7)^.

Hot flashes are among the most common side effects of ADT. They affect approximately 50%-80% of patients receiving ADT and are associated with decreased quality of life ^(1), (8), (9)^, making side effect management important. A prospective cohort study in the United States reported that ADT-induced hot flashes were more common in patients with prostate cancer who were younger and had a higher body mass index (BMI) ^(10)^. Conversely, a single-center retrospective study in the United Kingdom reported a lower incidence of hot flashes in patients with prostate cancer and concomitant ischemic heart disease ^(11)^. A randomized controlled trial conducted in Finland reported an association between beta-blocker use and a lower incidence of menopausal hot flashes in postmenopausal women ^(12)^. Nevertheless, the risk factors for ADT-induced hot flashes in Japanese patients with prostate cancer, including the effects of concomitant medications, have not been explored.

This study aimed to identify the risk factors for hot flashes associated with ADT by predicting their onset on the basis of the baseline characteristics of Japanese patients with prostate cancer.

## Materials and Methods

### Ethical considerations

This study was approved by the ethics review committees of the Federation of National Public Service Personnel Mutual Aid Associations of Hirakata Kohsai Hospital (approval number: 2024-015) and Kyoto Pharmaceutical University (approval number: E-00053). It was a non-invasive observational study without intervention and did not involve the use of human-derived samples. Therefore, informed consent was not required. However, an opt-out system was provided to ensure that the patients could withdraw their consent because the study used their medical records.

### Study procedures and participants

This was a single-center retrospective cohort study using medical records from the Department of Pharmacy, Federation of National Public Service Personnel Mutual Aid Associations Hirakata Kohsai Hospital. The study included patients who received luteinizing hormone-releasing hormone (LH-RH) agonists or antagonists at our hospital between January 2013 and December 2023. Patients younger than 18 years, those who initiated ADT at other institutions, those with communication difficulties, and those with insufficient interview records were excluded.

### Data collection

The demographic data of the participants collected at the initiation of LH-RH agonist or antagonist therapy included height, weight, BMI, and comorbid cardiovascular diseases, including heart failure, atrial fibrillation, ischemic heart disease, and hypertension. The prostate cancer-related factors included the initial prostate-specific antigen concentration, tumor, node, metastasis classification, Gleason score, prostate volume, combined androgen blockade therapy, and type of ADT. The concomitant medications investigated included cardiovascular drugs (renin-angiotensin-aldosterone system inhibitors, beta-blockers, nitrate drugs, and calcium channel blockers), steroids, antiepileptic drugs, herbal medicines, and antidepressants. The presence of hot flashes and their time of onset were evaluated on the basis of medical records documenting patient interviews conducted during LH-RH agonist or antagonist administration. No standardized questionnaires were used for this assessment.

### Statistical analysis

The patients were divided into hot flash onset and non-onset groups. Their characteristics were compared using Fisher’s exact and Mann-Whitney U tests. Multivariable logistic regression analysis was performed to identify factors associated with hot flash suppression. The presence of hot flashes was the dependent variable, and cardiovascular diseases and their treatments were the explanatory variables. The covariates included in the multivariate logistic regression analysis (age, BMI, cardiovascular diseases, and cardiovascular medications) were selected a priori on the basis of their clinical relevance and previous literature. Age and BMI were included because prior studies have suggested their association with the onset of hot flashes in patients receiving ADT ^(10), (11)^. Cardiovascular diseases and their therapeutic medications were included on the basis of reports indicating potential associations with vasomotor symptoms ^(11), (12)^. The type of ADT (LH-RH agonist vs. antagonist) was not included as a covariate because prior research has indicated no significant difference in the overall incidence of hot flashes between these two types ^(13)^. These variables were predetermined before data analysis to maintain the objectivity and reproducibility of the study. The variance inflation factor (VIF) was calculated for each variable to assess multicollinearity in the regression model. Statistical analyses were performed using EZR version 1.61 (based on R version 4.3.1) (Saitama Medical Center, Jichi Medical University, Saitama, Japan) ^(14)^. Statistical significance was set at p < 0.05.

## Results

### Patient selection

**Figure 1.**
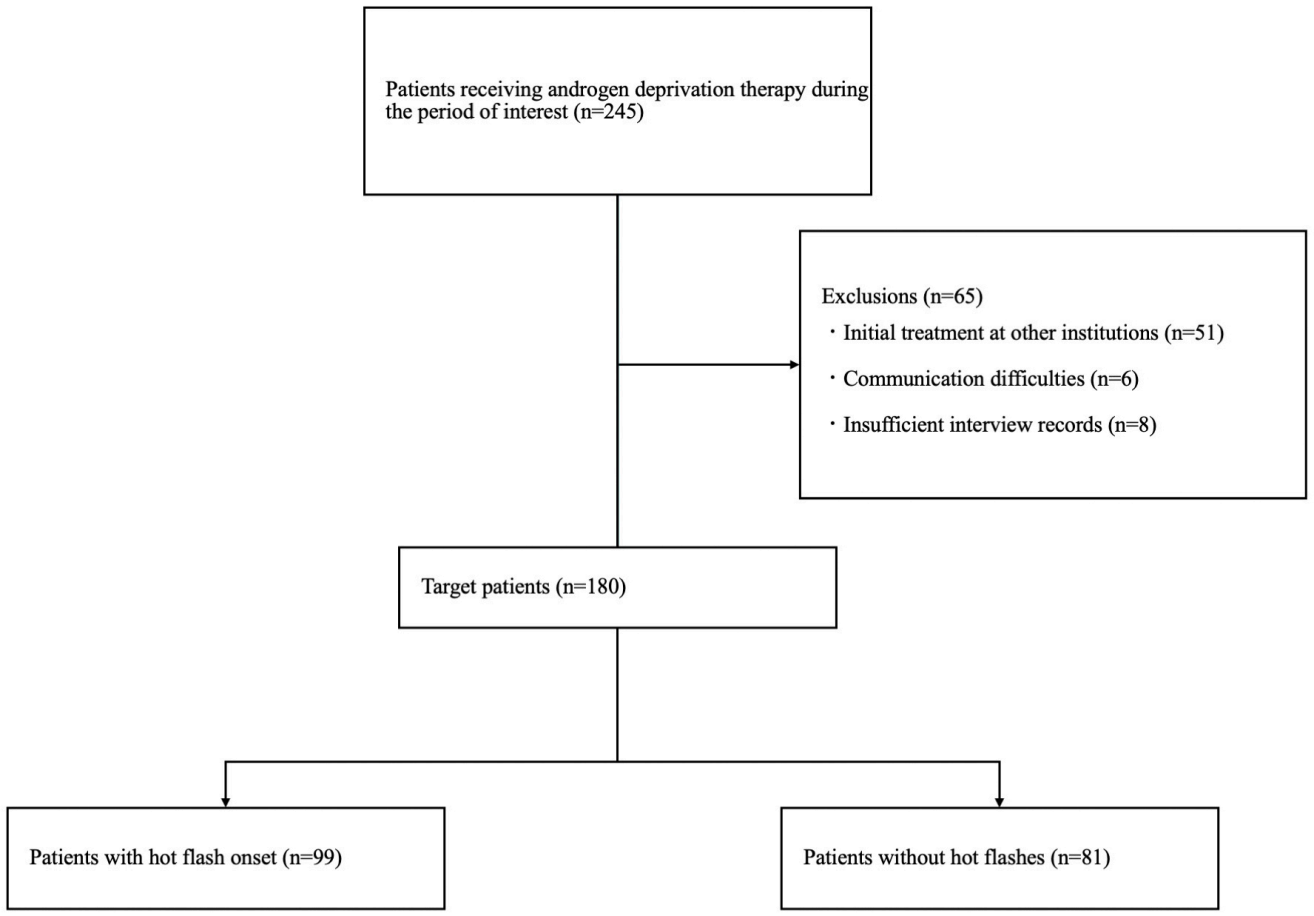
Flowchart of patient selection based on the medical records.

The electronic medical records of 245 patients who received LH-RH agonists or antagonists at our institution between January 2013 and December 2023 were reviewed. Fifty-one patients who had initiated treatment at other institutions, six with communication difficulties, and eight with insufficient interview records were excluded. The data of the remaining 180 patients were included in the final analysis (Figure 1).

### Patient characteristics

Table 1 lists the baseline characteristics based on the presence or absence of hot flashes. Of the 180 patients, 99 (55.0%) had hot flashes, whereas 81 (45.0%) did not. Those with hot flashes were significantly younger (median age, 76 vs. 77 years, p = 0.019) and had lower prevalences of atrial fibrillation (4.0% vs. 21.0%, p = 0.001) and beta-blocker use (11.1% vs. 32.1%, p = 0.001) than those without. The median observation period for ADT was significantly longer in patients with hot flashes than in those without (827 days [interquartile range (IQR): 331-1,760] vs. 333 days [IQR: 140-895], p < 0.001).

**Table 1.** Baseline Characteristic of Hot Flashes Onset and Non-Onset Groups.

Parameter	Hot flashes		Total (n = 180)	p-Value
	Onset (n = 99)	Non-onset (n = 81)		
Age, years	76 (70-79)	77 (74-82)	76 (72-80)	0.019
Height, cm	167.0 (163.0-170.0)	164.9 (160.0-167.9)	165.5 (162.0-169.9)	0.004
Weight, kg	67.0 (60.0-71.2)	60.0 (54.0-69.0)	64.4 (57.0-70.6)	0.003
BMI	23.3 (22.1-25.9)	22.7 (20.8-25.4)	23.1 (21.2-25.7)	0.069
Initial PSA, ng/mL	15.7 (8.2-51.0)	16.7 (8.3-44.7)	15.8 (8.3-47.0)	0.570
Prostate volume, mL	33.6 (22.9-47.7)	21.2 (25.0-43.8)	32.4 (24.2-45.2)	0.983
Smoking				
Yes	15 (15.3)	12 (15.0)	27 (15.2)	1.000
No	83 (84.7)	68 (85.0)	151 (84.8)	
Hormonal therapy				
LH-RH agonist	75 (75.8)	54 (66.7)	129 (71.7)	0.188
LH-RH antagonist	24 (24.2)	27 (33.3)	51 (28.3)	
Combined androgen blockade	92 (92.9)	71 (87.7)	163 (90.5)	0.212
TNM-based grouping				
N0M0	64 (64.6)	54 (66.7)	118 (65.6)	0.875
N1 or M1	35 (35.4)	27 (33.3)	62 (34.4)	
Gleason score	8 (7-9)	8 (7-9)	8 (7-9)	0.704
Comorbidity				
Heart failure	9 (9.1)	13 (16.0)	22 (12.2)	0.176
Ischemic heart disease	16 (16.2)	13 (16.0)	29 (16.1)	1.000
Hypertension	52 (52.5)	44 (54.3)	96 (53.3)	0.881
Atrial fibrillation	4 (4.0)	17 (21.0)	21 (11.7)	0.001
Diabetes	17 (17.2)	12 (14.8)	29 (16.1)	0.690
Drugs for cardiovascular diseases				
Beta-blockers	11 (11.1)	26 (32.1)	37 (20.6)	0.001
RAS inhibitors	40 (40.4)	37 (45.7)	77 (42.7)	0.545
Calcium channel blockers	47 (47.5)	37 (45.7)	84 (46.7)	0.881
Nitrate drugs	6 (6.1)	3 (3.7)	9 (5.0)	0.517
Other drugs				
Steroids	2 (2.0)	1 (1.2)	3 (1.7)	1.000
Anticonvulsants	1 (1.0)	0 (0)	1 (0.6)	1.000
Herbal medicines	4 (4.0)	2 (2.5)	6 (3.3)	0.692
Antidepressants	0 (0)	1 (1.2)	1 (0.6)	0.450
Dopamine agonists	0 (0)	0 (0)	0 (0)	-

Data are presented as n (%), median (IQR), or units as indicated in the table. Fisher’s exact test for categorical variables and Mann-Whitney U test for continuous variables. BMI: body mass index; Initial PSA: PSA level measured in a patient at the time of diagnosis or at the first clinical examination evaluation for prostate cancer; IQR: the range between the 25th and 75th percentiles of the data; LH-RH: luteinizing hormone-releasing hormone; RAS inhibitor: medications that block the renin-angiotensin system; TNM: tumor, node, metastasis.

### Risk factors for hot flashes

The results of the multivariate logistic regression analysis of the risk factors for hot flash onset are listed in Table 2. Multicollinearity was assessed using VIFs, and all explanatory variables showed VIF values <3.

**Table 2.** Multivariate Logistic Regression Analysis to Identify the Risk Factors for Hot Flashes.

Independent variables		Hot flashes	
		OR (95% CI)	P-value
Younger		1.056 (1.004-1.110)	0.034
High BMI		0.917 (0.820-1.020)	0.110
Cardiovascular disease	Heart failure	2.090 (0.560-7.820)	0.272
	Atrial fibrillation	0.257 (0.071-0.928)	0.038
Therapeutic drugs	Beta-blockers	0.265 (0.086-0.816)	0.021
	RAS inhibitors	0.826 (0.407-1.670)	0.595
	Calcium channel blockers	1.310 (0.660-2.610)	0.439
	Nitrate drugs	2.060 (0.434-9.750)	0.364

The variance inflation factor (VIF) was calculated for each variable to assess multicollinearity in the regression model. VIF < 3 for all explanatory variables. BMI: body mass index; CI: confidence interval; OR: odds ratio; RAS inhibitors: medications that block the renin-angiotensin system.

Younger age was significantly associated with increased odds of hot flashes (odds ratio [OR]: 1.056, 95% confidence interval [CI]: 1.004-1.110, p = 0.034). In contrast, atrial fibrillation (OR: 0.257, 95% CI: 0.071-0.928, p = 0.038) and beta-blocker therapy (OR: 0.265, 95% CI: 0.086-0.816, p = 0.021) were significantly associated with reduced risks of hot flashes. No significant associations were observed between hot flashes and BMI, history of heart failure, or the use of calcium channel blockers, renin-angiotensin-aldosterone system inhibitors, or nitrate drugs.

## Discussion

In this study, hot flashes were observed in 55.0% of the participants. We showed that younger Japanese patients with prostate cancer had a significantly higher risk of hot flash onset during ADT. Our results suggest that atrial fibrillation and beta-blockers may suppress ADT-related hot flash onset.

Previous studies conducted in the United States have reported that younger patients with prostate cancer may have a higher risk of hot flash onset ^(10)^, which is consistent with the findings of our study. The association between age and hot flashes is believed to be related to age-dependent changes in the hypothalamic-pituitary-gonadal axis ^(15)^. The hypothalamic-pituitary system may adapt to androgen deficiency in older adults after prolonged reductions in sex hormone concentrations ^(16)^. Older adults may have an attenuated response to the acute decrease in androgen concentrations induced by ADT. This may contribute to their lower risk of hot flash onset than that in younger adults.

A study from the United Kingdom reported a correlation between BMI and the severity of hot flashes in patients with prostate cancer receiving ADT ^(11)^. Obesity has been suggested to contribute to hot flashes through impaired heat dissipation associated with adiposity, increased sympathetic activity, and elevated inflammatory cytokines ^(17)^. The median BMI in that study was 28.0 (IQR: 24.4-31.0). However, we found no significant association between BMI and hot flashes in our Japanese cohort. The median BMI of the participants was 23.1 (IQR: 21.2-25.7), which is substantially lower than the values reported previously. This difference likely reflects disparities in body composition between Japanese and Western populations. Obesity may play a more prominent role in the onset of hot flashes in cohorts with a higher BMI. However, its influence appears limited in Japanese patients with prostate cancer, who have relatively low BMI.

Ischemic heart disease was associated with a reduced incidence of hot flashes in patients with prostate cancer who underwent ADT in a study from the United Kingdom ^(11)^. This was attributed, at least in part, to the potential suppressive effects of nitrate drugs, which are commonly prescribed to this population ^(11)^. However, we did not observe a significant association between ischemic heart disease and hot flashes. Several factors may explain this discrepancy. Only 9 (31.0%) of the 29 patients with ischemic heart disease in our study were receiving nitrate therapy. Direct comparison is difficult because the previous report did not provide data on nitrate drug use among patients with ischemic heart disease. Nitrate prescriptions may have been restricted owing to the risk of orthostatic hypotension in our Japanese participants, who were older and had smaller body habitus. These factors may have contributed to the lack of significant association in our study.

A key finding of this study is the significant association between beta-blocker use and hot flash suppression. Sex hormones typically induce the production of hypothalamic beta-endorphins and catechol estrogens. These compounds inhibit norepinephrine synthesis in the hypothalamus through negative feedback. Hot flashes occur after ADT reduces androgen production and the associated negative feedback ^(8)^. Beta-blockers have also been reported to alleviate hot flashes in postmenopausal women ^(12)^. However, to the best of our knowledge, no prior study has evaluated this association in Japanese patients with prostate cancer. Our findings provide novel insight into the relationship between beta-blocker use and ADT-related hot flashes. Beta-blockers are well-established and safe medications. Their potential suppressive effect on hot flashes may represent a promising drug repurposing opportunity. Future multicenter prospective trials are warranted to confirm these findings.

We also observed that atrial fibrillation was associated with a lower incidence of hot flashes. Multicollinearity was not observed in the multivariate logistic regression analysis (model VIF <3 for all explanatory variables). Atrial fibrillation and beta-blocker use were identified as independent protective factors. This suggests that atrial fibrillation may have an inherent suppressive effect on hot flashes. Atrial fibrillation is characterized by autonomic dysregulation, and alterations in sympathetic activity or catecholamine secretion may influence the onset of hot flashes. However, the precise mechanism is unclear, and further investigation is warranted.

This study has some limitations. First, it was a single-center retrospective study, and selection and information biases cannot be ruled out. The presence of hot flashes was determined on the basis of patient-reported data from interviews, and no standardized questionnaires were used. This may have introduced variability in symptom reporting. Second, the sample was relatively small, precluding a detailed evaluation of the type, dosage, and duration of beta-blocker therapy. Third, the observation period differed significantly between patients with and those without hot flashes, which may have influenced the detection of hot flashes. In addition, hormonal data were not systematically collected, given routine hormonal monitoring during ADT was not standard practice at our institution. Future prospective studies with systematic hormone measurements and that account for differences in observation periods are warranted to better evaluate the associations between hormonal status, beta-blocker use, and hot flash onset. Despite these limitations, our study identified risk factors for ADT-related hot flashes in Japanese patients with prostate cancer. These are underexplored, and the findings of this study represent clinically meaningful insights.

Younger age is a risk factor for hot flashes in Japanese patients with prostate cancer receiving ADT, and they require careful monitoring. Conversely, atrial fibrillation and beta-blocker therapy may contribute to suppressing the onset of hot flashes.

Given these findings, beta-blockers may be effective for the prevention or treatment of hot flashes. Further investigation is warranted from the perspective of drug repurposing, particularly prospective trials evaluating beta-blocker efficacy for preventing ADT-related hot flashes, optimal dosing strategies, and comparative studies of different beta-blocker subtypes.

## Article Information

### Author Contributions

Momoko Sawada: Substantial contributions to the conception, planning, and design of the study; supervised data collection; contributed to data analysis and interpretation; participated in drafting and critically reviewing the manuscript for important intellectual content; gave final approval of the version to be published; and agreed to be accountable for all aspects of the work. Ryo Inose: Substantial contributions to the conception, design, and planning of the study; contributed to data analysis and interpretation; participated in critically reviewing the manuscript for important intellectual content; gave final approval of the version to be published; and agreed to be accountable for all aspects of the work. Kosuke Okasho: Contributed to data analysis and interpretation; participated in critically reviewing the manuscript for important intellectual content; gave final approval of the version to be published; and agreed to be accountable for all aspects of the work. Yuichi Muraki: Substantial contributions to the study design and planning; contributed to data analysis and interpretation; participated in drafting and critically reviewing the manuscript for important intellectual content; supervised protocol compliance at Kyoto Pharmaceutical University; gave final approval of the version to be published; and agreed to be accountable for all aspects of the work. Shuji Kono: Supervised data collection; contributed to data interpretation; participated in critically reviewing the manuscript for important intellectual content; gave final approval of the version to be published; and agreed to be accountable for all aspects of the work.

### Conflicts of Interest

Ryo Inose received funding for the commissioned research from the Kowa Company, Ltd. However, this study was not directly funded. Yuichi Muraki received grants from Pfizer Japan Inc.; Kowa Company Ltd.; the Japan Pharmaceutical Association; the Japan Society for the Promotion of Science; and the Ministry of Health, Labour and Welfare. Yuichi Muraki is also a board member of the Japanese Society of Pharmaceutical Health Care and Sciences and the Japanese Society for Infection Prevention and Control, and a committee member of the AMR Clinical Reference Center. However, this study was not directly funded. The other authors have no conflicts of interest to declare.

### IRB Approval Code and Name of the Institution

Federation of National Public Service Personnel Mutual Aid Associations Hirakata Kohsai Hospital (Approval No. 2024-015); Kyoto Pharmaceutical University (Approval No. E-00053).
